# Chromosome number variation of the Italian endemic vascular flora. State-of-the-art, gaps in knowledge and evidence for an exponential relationship among even ploidy levels

**DOI:** 10.3897/CompCytogen.v6i2.3107

**Published:** 2012-05-07

**Authors:** Gianni Bedini, Fabio Garbari, Lorenzo Peruzzi

**Affiliations:** 1 Dipartimento di Biologia, Unità di Botanica generale e sistematica, Università di Pisa, via Luca Ghini 5, 56126, Pisa, Italy

**Keywords:** B-chromosomes, cytotaxonomy, endemics, evolution, Italy, polyploidy

## Abstract

The Italian endemic vascular flora is composed of 1,286 specific and subspecific taxa. From the critical analysis of “Chrobase.it”, 711 of them (about 55%) have been studied from a karyological point of view. These taxa belong to 52 out of 56 families and 204 out of 284 genera. These data suggest that endemic species are more studied than the flora as a whole. Mean chromosome number for Italian endemics is 2n = 30.68 ± 20.27 (median: 2n = 26, mode: 2*n* = 18). These values are very close to those known for the whole flora. Similar variation ranges, among endemics and species with wider distribution, are likely to reflect similar evolutionary trends. Known chromosome numbers in Italian endemics range from 2n = 8 to 2n = 182. About 9% of taxa show more than one cytotype and the frequency of Bs in the Italian endemic vascular flora is 3.3%. These values are slightly smaller compared with the whole Italian flora. Finally, for the basic chromosome numbers x = 7, 8, 9, the proportion of diploids (2n = 2*x*) to even polyploids (2*n* = 4x, 6x, 8x and 10x) can be described by the exponential function f(p) = e^(5.539 – 0.637p)^ (R^2^ = 0.984).

## Introduction

The number of chromosome count databases, either hard-printed or online, matches current research trends, and attests to the usefulness of chromosome data in current taxonomic, genetic and cytogeographic research (Del Prete et al. 1999; Guerra 2008; Stuessy 2009; Bedini et al. 2012a, b). Recent studies (Peruzzi et al. 2011; Bedini et al. 2012a, b) exemplified the potentialities of such databases in inferring evolutionary and cytogeographic relationships. Italian vascular flora coverage, in terms of karyological knowledge, is about 35% (Peruzzi et al. 2011; Bedini et al. 2012a, b).

The Italian endemic vascular flora, according to on-going research carried out in collaboration with CRFA (Centro di Ricerca per la Flora dell’Appennino) of Barisciano (L’Aquila, Italy), comprises 1,286 specific and subspecific taxa(L. Peruzzi, F. Bartolucci and F. Conti, unpublished data), including those species which eventually occur also in Corse (France). The present work aims to summarize the karyological knowledge focusing on the endemic component of Italian vascular flora, extracted from the online database “Chrobase.it” (Bedini et al. 2010 onwards).

## Methods

### Source of chromosome data

Data about chromosome numbers (*n* and/or 2*n*) and B-chromosomes about Italian vascular flora are stored in the online database “Chrobase.it” (Bedini et al. 2010 onwards). Up to March 20, 2012, the database consisted of 7,560 records, derived from 1,364 literature references, dating back to 1925 (Garbari et al. 2012). They refer to 3,035 accepted taxa, according to the nomenclature of Conti et al. (2005, 2007). Data for endemic taxa were obtained by querying “Chrobase.it” with the list of endemics and relative synonyms (the complete list of endemics, including those lacking karyological knowledge, is available at request from the authors). The number of available counts and taxa were calculated for Italy as a whole, for each of the 20 Italian administrative regions, and for each family and genus. Counts in multiple copy (i.e., the same chromosome number for the same species) were excluded from further analyses, while different chromosome counts for the same taxon (i.e. cytotypes) were retained. Any n count was transformed to 2n and then included in the dataset. 25 counts, referring to 25 different taxa, obtained from Corsican populations were also included, given the high number of endemics shared by Sardinia, Corse and Tuscan Archipelago. From 24 of these units no counts were available for the Italian territory.

Concerning the list of endemics, for the difficult and critical genera *Hieracium* L. (1753) and *Pilosella* Vaill. (1754) (Asteraceae) the subspecies rank was not considered.

### Analysis of data

Mean (± standard deviation), median, modal chromosome number, and frequencies (histograms) were calculated for the entire dataset of Italian vascular flora endemics. Frequency and mean number (± standard deviation) of B-chromosomes for the whole dataset and for each genus were also calculated. The frequencies of basic chromosome numbers (x) in those complements (2n) where more than one basic number can occur were obtained by a taxon by taxon screening of relevant karyological literature quoted in Chrobase.it (Bedini et al. 2010 onwards). Frequencies of even ploidy levels from 2*x* to 10*x*, in the three most frequent basic numbers x = 7, x = 8 and x = 9, were arranged in a linear plot, and their relationships between ploidy levels was tested by means of linear and non linear models based on least-squares estimates in R software (www.r-project.org; Rossiter 2009): lm function was used to fit a straight line, nls function was used for power, logarithmic, and exponential curves. Odd ploidy levels were not considered since they were very rare in our data set, with the only exception of 2n = 27 (see over).

## Results

**Figure 1.**
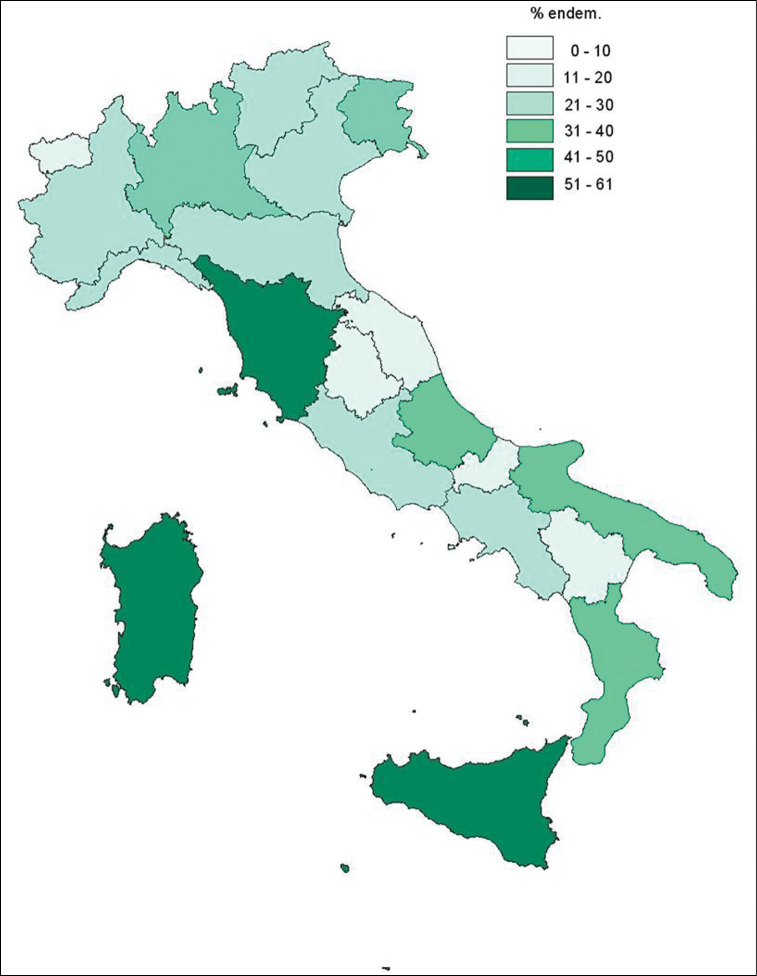
Map of Italian regions, showing the % endem. of Italian vascular flora karyologically studied with respect to the Italian endemics growing in each region (derived from Conti et al. 2005).

Chromosome counts are available for 711 out of the 1,286 (ca. 55%) currently accepted specific and infraspecific endemic taxa, resulting in 839 different cytotypes; they are representative of 204 out of the 284 genera (72%) – and 52 out of the 56 families (91%) – encompassing the endemic taxa. The geographic distribution of counts is shown in Fig. 1. The most intensely investigated regions are Sicily, Tuscany and Sardinia, where more than half of the Italian endemic flora growing in the respective territories was karyologically studied. Table 1 shows the number of taxa studied for each region.

**Table 1.** Number of karyologically studied Italian endemic vascular flora for each region. The sequence of regions is the same as the one used in Conti et al. (2005).

**Region**	**No. taxa studied**
Valle d’Aosta	0
Piedmont	7
Lombardy	13
Trentino-Alto Adige	9
Veneto	10
Friuli-Venezia Giulia	6
Liguria	9
Emilia-Romagna	9
Tuscany	89
Marche	10
Umbria	9
Latium	26
Abruzzo	61
Molise	2
Campania	26
Apulia	33
Basilicata	10
Calabria	75
Sicily	193
Sardinia	156

**Figure 2.**
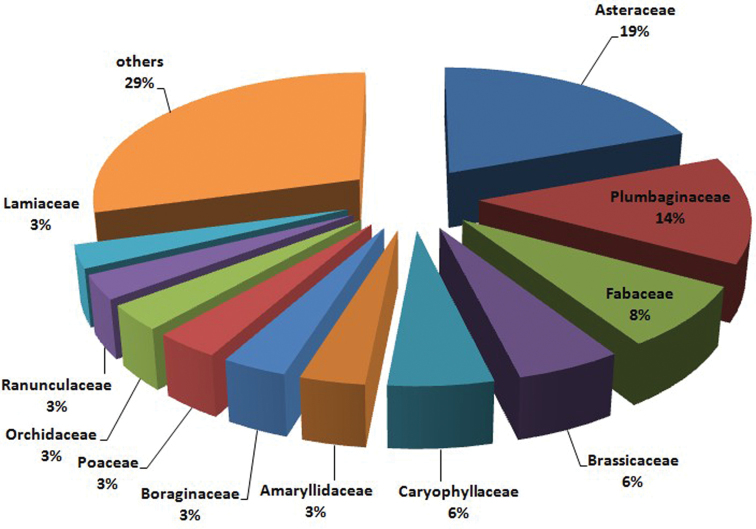
Pie plot showing the distribution of karyologically studied Italian endemic taxa across families. Families with less than 20 studied taxa were merged.

The distribution of the 711 taxa across families is shown in Fig. 2. The most represented families are Asteraceae (19%), Plumbaginaceae (14%), Fabaceae (8%), Brassicaceae and Caryophyllaceae (6%). For all the endemics from Rosaceae (26 taxa), Thymelaeaceae (2 taxa), Aspleniaceae, Berberidaceae, Cannabaceae, Convolvulaceae, Cucurbitaceae, Oleaceae, Papaveraceae and Pinaceae (1 taxon each), no karyological data is available in the literature.

For 92 genera, of which 7 have ≥ 5 endemics, 100% of Italian endemics was covered (Table 2). Other 29 genera, hosting ≥ 5 endemics, showed coverage ranging from 93% in *Genista* L. (1753) to only 10% in *Hieracium*. The highest number of studied taxa occurs in the genera *Limonium* Mill. (1754) (84 *taxa*), *Centaurea* L. (1753) (46 taxa) and *Genista* (26 taxa).

**Table 2.** Most karyologically studied genera of Italian endemics (≥ 5 taxa). They are arranged firstly according their decreasing % of coverage, secondly their number of taxa studied and, thirdly, alphabetically.

**Genus**	**No. taxa studied**	**Coverage**
*Anchusa* L. (1753)	7	100%
*Ornithogalum* L. (1753)	7	100%
*Crocus* L. (1753)	6	100%
*Santolina* L. (1753)	6	100%
*Sesleria* Scop. (1760)	6	100%
*Stachys* L. (1753)	6	100%
*Onosma* L. (1762)	5	100%
*Genista* L. (1753)	26	93%
*Allium* L. (1753)	18	90%
*Viola* L. (1753)	17	85%
*Limonium* Mill. (1754)	83	84%
*Cardamine* L. (1753)	5	83%
*Erysimum* L. (1753)	5	83%
*Iris* L. (1753)	8	80%
*Campanula* L. (1753)	14	77%
*Biscutella* L. (1753)	10	77%
*Anthemis* L. (1753)	8	75%
*Pinguicula* L. (1753)	6	75%
*Brassica* L. (1753)	8	73%
*Cerastium* L. (1753)	8	73%
*Euphorbia* L. (1753)	10	71%
*Jacobaea* Mill. (1754)	5	71%
*Armeria* Willd. (1809)	11	69%
*Astragalus* L. (1753)	9	69%
*Senecio* L. (1753)	6	67%
*Centaurea* L. (1753)	45	66%
*Helichrysum* Mill. (1754)	6	60%
*Taraxacum* F.W. Wigg. (1780)	9	53%
*Festuca* L. (1753)	8	53%
*Asperula* L. (1753)	6	50%
*Ranunculus* L. (1753)	16	48%
*Saxifraga* L. (1753)	7	47%
*Ophrys* L. (1753)	14	38%
*Silene* L. (1753)	10	38%
*Dianthus* L. (1753)	8	30%
*Hieracium* L. (1753)	5	10%

Genera with 100% karyological coverage but with less than 5 Italian endemics: *Cynoglossum* L. (1753) (4), *Erodium* L’Hér. (1789) (3), *Gagea* Salisb. (1806) (3), *Plantago* L. (1753) (3), *Arenaria* L. (1753) (2), *Athamanta* L. (1753) (2), *Borago* L. (1753) (2), *Buphthalmum* L. (1753) (2), *Globularia* L. (1753) (2), *Hypericum* L. (1753) (2), *Isoëtes* L. (1753) (2), *Moltkia* Lehm. (1817) (2), *Muscari* Mill. (1754) (2), *Oncostema* Raf. (1837) (2), *Orchis* L. (1753) (2), *Paeonia* L. (1753) (2), *Ptilostemon* Cass. (1816) (2), *Pulmonaria* L. (1753) (2), *Rhaponticoides* Vaill. (1754) (2), *Ribes* L. (1753) (2), *Soldanella* L. (1753) (2), *Thapsia* L. (1753) (2), *Tragopogon* L. (1753) (2), *Veronica* L. (1753) (2), *Acer* L. (1753) (1), *Adonis* L. (1753) (1), *Adoxa* L. (1753) (1), *Ajuga* L. (1753) (1), *Anemonoides* Mill. (1754) (1), *Arum* L. (1753) (1), *Arundo* L. (1753) (1), *Bellium* L. (1771) (1), *Bituminaria* Fabr. (1759) (1), *Calendula* L. (1753) (1), *Callianthemum* C.A. Mey. (1830) (1), *Chiliadenus* Cass. (1825) (1), *Coristospermum* Bertol, (1838) (1), *Cryptotaenia* DC. (1829) (1), *Digitalis* L. (1753) (1), *Diplotaxis* DC. (1821) (1), *Drymochloa* Holub (1984) (1), *Echinops* L. (1753) (1), *Edraianthus* (A. DC.) A. DC. (1839) (1), *Erucastrum* C. Presl (1826) (1), *Ferula* L. (1753) (1), *Goniolimon* Boiss. (1848) (1), *Jasione* L. (1753) (1), *Jurinea* Cass. (1821) (1), *Klasea* Cass. (1825) (1), *Lactuca* L. (1753) (1), *Lagurus* L. (1753) (1), *Lamium* L. (1753) (1), *Lamyropsis* (Kharadze) Dittrich (1971) (1), *Leucojum* L. (1753) (1), *Limodorum* Boehm. (1760) (1), *Mentha* L. (1753) (1), *Morisia* J. Gay (1832) (1), *Nananthea* DC. (1838) (1), *Nepeta* L. (1753) (1), *Nigritella* Rich. (1817) (1), *Oenanthe* L. (1753) (1), *Oxytropis* DC. (1802) (1), *Petagnaea* Caruel (1894) (1), *Phleum* L. (1753) (1), *Pimpinella* L. (1753) (1), *Plagius* DC. (1838) (1), *Ptilotrichum* C.A. Mey. (1831) (1), *Prospero* Salisb. (1866) (1), *Pseudoscabiosa* Davesa (1984) (1), *Ptychotis* W.D.J. Koch (1924) (1), *Quercus* L. (1753) (1), *Retama* Raf. (1838) (1), *Rhizobotrya* Tausch (1836) (1), *Ruta* L. (1753) (1), *Salicornia* L. (1753) (1), *Sideritis* L. (1753) (1), *Solenanthus* Ledeb. (1829) (1), *Solidago* L. (1753) (1), *Symphytum* L. (1753) (1), *Teucrium* L. (1753) (1), *Trachelium* L. (1753) (1), *Tripolium* Nees (1832) (1), *Urtica* L. (1753) (1), *Vinca* L. (1753) (1), *Zelkova* Spach (1841) (1).

Chromosome numbers range from 2n = 8, reported in 8 endemic taxa [*Bellevalia dubia* (Guss.) Schult. & Schult. f. (1830) s.s., *Crepis vesicaria* subsp. *hyemalis* (Biv.) Babc. (1941), *Crocus etruscus* Parl. (1860), *Crocus ilvensis* Peruzzi & Carta (2011), *Crocus siculus* Tineo (1832), *Hypochaeris robertia* (Sch. Bip.) Fiori (1910), *Leontodon anomalus* Ball (1850) and *Leontodon intermedius* Porta (1879)], to 2n = 180, 182, reported for *Colchicum gonarei* Camarda (1978).

**Figure 3.**
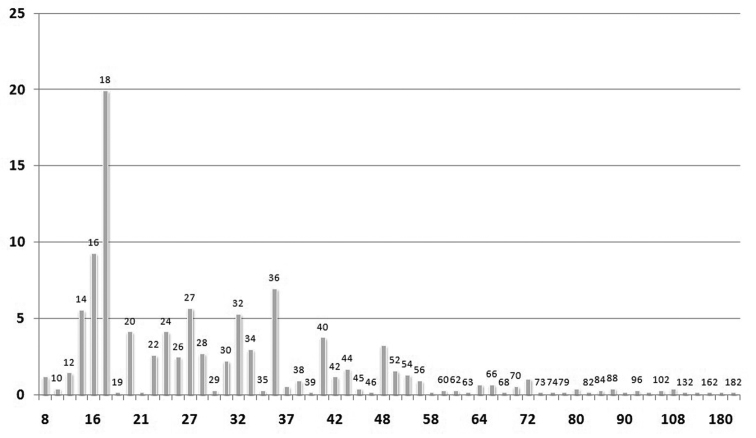
Histograms showing the percentage frequencies (y-axis) of 2n chromosome numbers (x-axis) known for the Italian endemic vascular flora.

The mean chromosome number is 2n = 30.68 ± 20.27, with median 2n = 26 and mode 2n = 18. Fig. 3 shows the most frequent chromosome numbers in the whole dataset of the Italian endemics. The six most frequent chromosome numbers are 2n = 14, 2n = 16, 2n = 18, 2n = 27, 2n = 32 and 2n = 36. Taken together, they account for more than one half (51%) of all the counts available.

Concerning chromosome complements where more than one basic number can occur, the most heterogeneous, among those up to 2n= 72 (Table 3), are: 2n = 24, which in most cases represents diploids with x = 12, but also triploids with x = 8 and rarely tetraploids with x = 6; 2n = 36, which in most cases represents tetraploids with x = 9, then diploids with x = 18 and rarely triploids with x = 12; 2n = 40, with tetraploids, pentaploids and diploids; 2n = 48, representing mostly by diploids with x = 24, but also tetraploids with x = 12 and hexaploids with x = 8; 2n = 56, representing mostly tetraploids with x = 14, but also diploids with x = 28 and octoploids with x = 7. The most frequent chromosome numbers, not reported in Table 3, include only diploids (2n = 2x = 14, 2n = 2x = 18) or triploids (2n = 3x = 27). When we apply these observations to the six most frequent chromosome numbers 2n = 14, 2n = 16 and 2n = 18, 2n = 27, 2n = 32 and 2n = 36, pooled together, we obtain 73.1% diploids, 15.9% tetraploids and 11% triploids.

**Table 3.** Chromosome complements up to 2n = 72, where more than one basic number (x) can occur, and respective x frequencies. The frequencies were obtained by a taxon-per-taxon literature screening. Higher chromosome numbers (very rare, in our dataset) were not considered.

**2n**	**Basic chromosome numbers (x)**												
	**4**	**5**	**6**	**7**	**8**	**9**	**10**	**12**	**14**	**16**	**18**	**20**	**21**
**8**	100%												
**12**			100%										
**16**	2.8%				97.2%								
**20**		3.1%					96.9%						
**24**			4.2%		20.8%			71%					
**28**				51%					49%				
**32**					71.9%					28.1%			
**36**						62.8%		2.3%			34.9%		
**40**					20.7%		62%					17.3%	
**42**				22%					11%				67%
	**6**	**7**	**8**	**9**	**11**	**12**	**13**	**14**	**18**	**22**	**28**	**32**	
**44**					92.3%					7.7%			
**48**			12%			88%							
**54**				90%					10%				
**56**		25%	12.5%					37.5%			25%		
**64**			80%									20%	
**70**		75%						25%					
**72**	12.5%			87.%									

**Figure 4.**
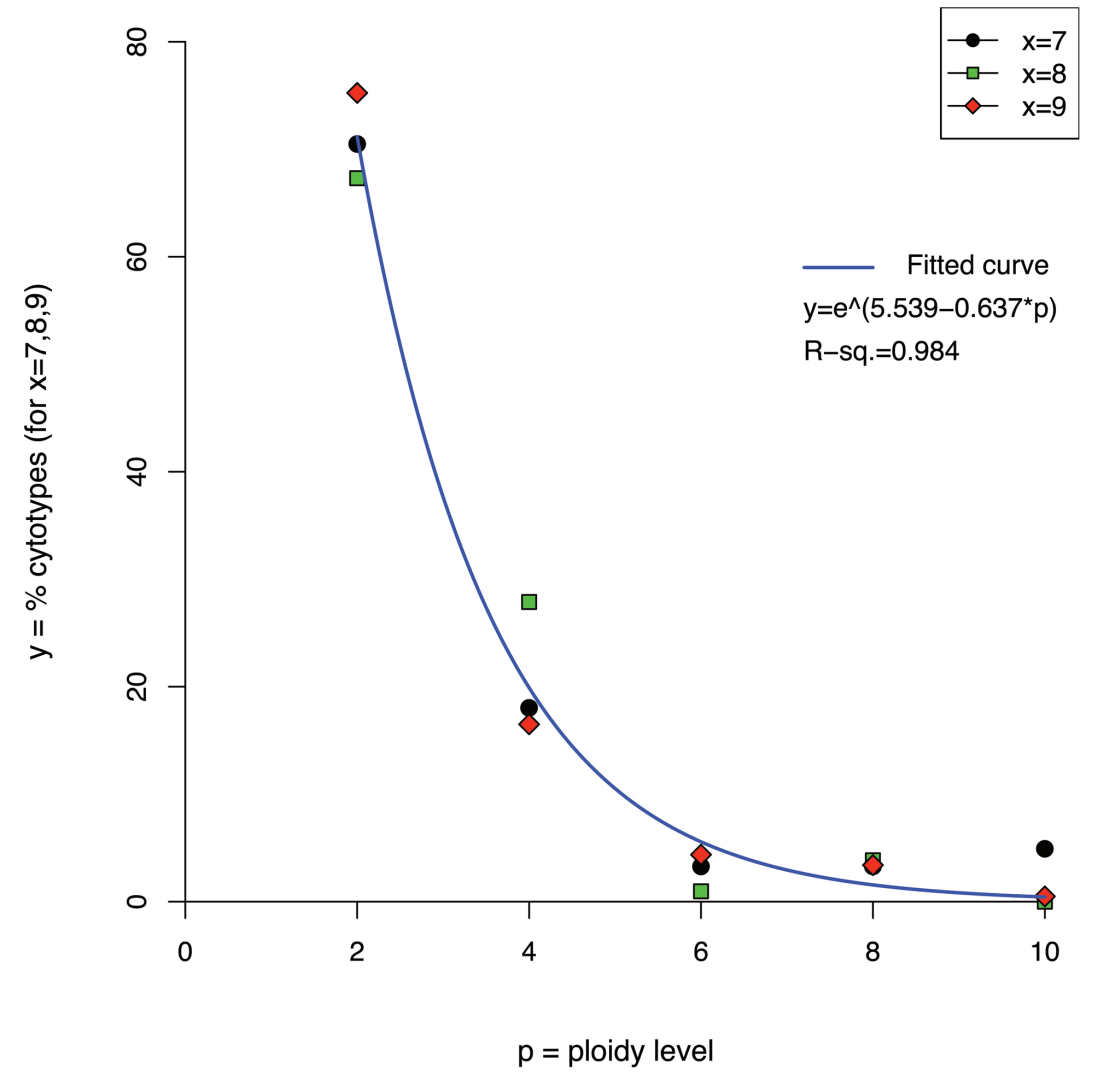
Plot showing the percentage frequencies (y-axis) of even ploidy levels from 2x to 10x (p-axis), for three frequent basic chromosome numbers (x = 7, x = 8 and x = 9) and the curve fitted to the data points by nonlinear least-square estimate.

The relationships among the different even ploidy levels, within each considered basic chromosome number (x = 7, x = 8, x = 9), was best described by an exponential function (Table 4). The power function provided the second-best fit, with a slightly higher RSS, followed by the logarithmic and linear functions, whose RSS is higher by an order of magnitude. Hence, diploids are much more frequent than polyploids, and frequency gradually decreases with increasing levels of polyploidy (Fig. 4).

**Table 4.** Goodness of fit of different models. The coefficients (a, b) matching the least-squares estimates are given for each model. RSS = Residual Sum of Squares. **= significant at 0.01 level.

**formula**	**coef. a**	**coef. b**	**RSS**	**R-squared**
y = a*x + b	7.786	66.714	3353.531	0.6844**
y = a*x^b	297.600	2.060	235.400	0.9780**
y = a + b*log(x)	91.940	-43.580	1422.000	0.8660**
y = e^(a + b*x)	5.539	-0.637	170.400	0.9840**

More than one cytotype was shown by 65 out of 711 taxa, with a maximum number of seven in *Crocus minimus* DC. (1804) (2n = 24, 25, 26, 27, 28, 29, 30); six in *Ornithogalum etruscum* Parl. (1857) subsp. *etruscum* (Asparagaceae): 2n = 72, 73, 74, 79, 90, 108 and in *Genista sulcitana* Vals. (1986) (Fabaceae): 2n = 18 + 0–2B, 27 + 0–2B; in 12 taxa, the variation is due to the possible presence of B-chromosomes.

B-chromosomes occur in 16/711 taxa and in 24 cytotypes (3.3% of the dataset), grouped in 10/284 genera (3.5%), 9/56 families (16%). Among the taxa showing B-chromosomes, their mean number is 1.73 ± 0.91, mode = 1 and median = 1.5. The highest number of B-chromosomes is 4, from a single accession of *Agrostis monteluccii* (Selvi) Banfi (2005) (Poaceae) and a single accession of *Rhinanthus wettsteinii* (Sterneck) Soó (1929) (Orobanchaceae). Table 5 shows the families and genera involved, with respective number of taxa showing B-chromosomes. Many records are concentrated in the genus *Genista*.

**Table 5.** Families and genera showing B-chromosomes in Italian vascular flora endemics, with the respective number of taxa (cytotypes), and the range of Bs.

Family	Genus	No. cytotypes with B	range of Bs
Fabaceae	*Genista* L. (1753) (5 taxa)	10	1–2
Poaceae	*Agrostis* L. (1753)	4	1–4
Asparagaceae	*Ornithogalum* L. (1753)	2	1, 3
Boraginaceae	*Onosma* L. (1762)	2	1
Boraginaceae	*Pulmonaria* L. (1753)	1	2
Lamiaceae	*Scutellaria* L. (1753)	1	2
Orchidaceae	*Orchis* L. (1753)	1	1
Orobanchaceae	*Rhinanthus* L. (1753)	1	4
Primulaceae	*Primula* L. (1753)	1	1
Ranunculaceae	*Ranunculus* L. (1753)	1	1

## Discussion

The taxonomic and geographic distribution of karyological knowledge in Italian endemics closely parallels that dealing with the whole Italian vascular flora (Bedini et al. 2012a). However, according to our data, Italian endemic species are significantly more studied than the whole flora, whose karyological coverage is about 35% (Bedini et al. 2012a). Central tendencies values of endemics (mean, median, mode) are very close to those known for the whole Italian flora (2n = 30.52 ± 22.09, median: 2n = 24, mode: 2n = 18) (Bedini et al. 2012a). This accounts for similar variation ranges among endemics and species with wider distribution and, probably, similar evolutionary trends. The frequency of B-chromosomes in endemics is on the contrary slightly smaller with respect to the whole Italian flora (Bedini et al. 2012a), but still much higher in comparison to other geographical areas with exceptionally high rate of endemism, such as for instance New Zealand (Peruzzi et al. 2011). Among the taxa not yet studied, we can point out an otherwise well known species such as *Abies nebrodensis* (Lojac.) Mattei (1908), the whole endemic component of several families (i.e. Rosaceae), and many species of critical Asteraceae genera (*Centaurea*, *Hieracium*).

The precise relationship [exponential function f(p) = e^(5.539 – 0.637p)^ (R^2^ = 0.984)] found among even ploidy levels from 2*x* to 10*x* within the frequent basic chromosome numbers x = 7, x = 8 and x = 9 was never reported before in literature, as far as we are aware (see also Levin 2002 for a recent review on chromosomal changes in plant evolution). At evidence, it seems that higher the (even) ploidy level, much lower is its frequency of occurrence. This could imply a sort of evolutionary constraint avoiding high ploidy levels. This point certainly deserves further investigations. Chromosome number evolution models take into account the fixation rates of polyploids at population level (Ramsey and Schemske 1998), or the reconstruction of ancestral chromosome numbers and the expected number of polyploidization events and single chromosome changes that occurred along a phylogeny (Mayrose et al. 2010; Cusimano et al. 2012). However, the latter works may suffer from a bias toward high ancestral chromosome number estimation (Guerra 2012). In any case, no attempt has ever been made to model the ratio of different even ploidy levels on a whole flora, albeit a similar work was recently done for Polish angiosperms (Gacek et al. 2011). The latter authors, however, mainly grossly estimated ploidy levels across their dataset by means of pre-established threshold numbers.

Odd ploidy levels are generally very rare in our dataset, with the noteworthy exception of triploids with 2n = 27. As already evidenced by Bedini et al. (2012a), this is due to a high number of (endemic) apomictic taxa within the genera *Hieracium* (Asteraceae) and *Limonium* (Plumbaginaceae).

The meaning of the precise relationship found in this work must be clarified by further analyses on large datasets of chromosome counts (e.g. PhytoKaryon; Bareka et al. 2008). If confirmed, it might provide evolutionary cytogenetic research with new insights as regards the frequency of polyploidization in vascular plants and its biological implications.

## Conclusions

Despite the efforts of Italian and foreign botanists in studying the endemic flora, the data here highlighted clearly show how much work is still to be done, concerning the karyological knowledge of Italian endemics. However, we were able to summarize the up-to-date knowledge, which accounts for more than one half of the endemic flora, and to suggest that these species likely followed karyological evolutionary processes similar to the whole flora. Moreover, as far as we are aware, it is the first time that a precise quantitative relationship between (even) ploidy levels is shown to occur. We demonstrated indeed that, for the frequent basic chromosome numbers x = 7, x = 8 and x = 9 the diploids dominate and are related to higher even ploidy levels by an exponential relationship. In our mind, this intriguing phenomenon opens a new line of investigation in cytogenetics, aimed to clarify the evolutionary mechanisms giving rise to these constant relationships among increasing even ploidy levels.
